# Our Best Friends: How Dogs Alter Indoor Air Quality

**DOI:** 10.1021/acs.est.5c13324

**Published:** 2026-02-02

**Authors:** Shen Yang, Nijing Wang, Tatjana Arnoldi-Meadows, Gabriel Bekö, Meixia Zhang, Marouane Merizak, Pawel Wargocki, Jonathan Williams, Martin Täubel, Dusan Licina

**Affiliations:** † Human-Oriented Built Environment Laboratory, School of Architecture, Civil and Environmental Engineering, 27218 École Polytechnique Fédérale de Lausanne (EPFL), 1015 Lausanne, Switzerland; ‡ School of Architecture, Southeast University, 210096 Nanjing, China; § 28309 Max Planck Institute for Chemistry, Hahn-Meitner Weg 1, 55128 Mainz, Germany; ∥ International Centre for Indoor Environment and Energy, Department of Environmental and Resource Engineering, 5205 Technical University of Denmark, 2800 Kongens Lyngby, Denmark; ⊥ Lifestyles and Living Environments, Department Public Health, 3837 Finnish Institute for Health and Welfare, P.O. Box 95, 70701 Kuopio, Finland; # Department of Civil Engineering, School of Engineering, Aalto University, P.O. Box 12100, 00076 Espoo, Finland

**Keywords:** pet, emission, built environment, airborne pollutant

## Abstract

Dogs are dynamic contributors to the indoor environment, yet their impact on air quality remains largely unexplored, warranting a comprehensive assessment of their pollutant emissions. This study characterized chemical, particulate, and microbial emissions from small and big dogs. Big dogs emitted CO_2_, NH_3_, fungi, and bacteria at considerably higher rates than small dogs, whereas their emissions of 1–10 μm particles were similar. With ozone present, all dogs contributed to the formation of nanocluster aerosols (1–3 nm) and ozonized volatile organic compound (VOC) products, likely from human skin oil transfer through petting. With ozone present, nanocluster aerosols (1–3 nm) were observed during dog experiments, likely reflecting ozone reactions with human-derived skin lipids transferred onto dog fur. Relative to a seated adult, big dogs emitted less ozonized products, comparable CO_2_ and NH_3_, more >5 μm coarse particles (fewer 2–5 μm particles), 2–4× more bacteria and fungi, and showed compound-specific differences in VOCs: while some species exhibited a strongly elevated dog-to-human ratio (with one up to 15×), others were not pronounced when dogs were present. Our findings highlight dogs as significant indoor emission sources and contributors to indoor air chemistry and microbial transport, with implications for air quality and exposure assessment.

## Graphical abstract

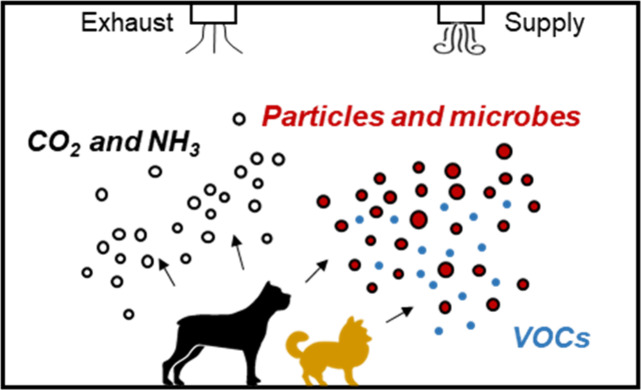

## Introduction

Dogs and humans share a long history. Indeed, it is claimed that dogs are the first domesticated animal.^1^ While canine companionship is believed to actively support mental well-being,^2^ its impact on human physical health remains unclear. Some studies suggest that having a dog in the household enhances physiological health, especially in children, where early exposure to associated microbes may lead to lower asthma risk and respiratory tract illness.^3–7^ Despite the mixed evidence regarding a protective effect of dog ownership against asthma and allergies,^8^ elevated risk is typically observed only among sensitized individuals. Understanding these health effects requires a dedicated examination of the pollutants that dogs emit and their impact on indoor exposures—an area that remains largely unexplored.

As mammalian vertebrates, dogs and humans share certain physiological processes, which may result in similarities in pollutant emissions. Earlier research has established humans as prodigious sources of indoor gaseous and particulate pollutants, as well as of microbes. Some of these emissions arise from endogenous processes: for instance, humans release carbon dioxide (CO_2_) and a suite of volatile organic compounds (VOCs) through respiration,^9–11^ and ammonia (NH_3_) through the skin.^12,13^ Additionally, skin shedding contributes significantly to indoor particulates and microbial matter.^14–16^ Other emissions are exogenous; for example, interactions between ozone and skin oils produce various VOCs and ultrafine particles.^17–20^ Human skin and clothing also serve as carriers, transporting gases, particles and microbes between environments.^21,22^

Building on our understanding of human emissions, we hypothesize that dogs may exhibit similar emission behaviors. However, the physiological differences between dogs and humans—such as variations in hair coverage, skin lipids, and sweat glands—are likely to result in distinct emission profiles. Surprisingly, despite the ubiquity of dogs in homes worldwide and their frequent physical interaction with humans and surfaces, no prior studies have systematically quantified the chemical, particulate, and microbial emissions from dogs. While dogs are known to affect indoor microbial environment, a simultaneous and controlled quantification of their chemical, particulate and microbial emissions is lacking. This represents a critical gap in our understanding of indoor air quality and exposure.

To address this gap, we conducted a pioneering experimental study to quantify the gas-phase and particulate emissions from dogs under controlled conditions. Using a state-of-the-art climate chamber, we measured a comprehensive suite of pollutants, including CO_2_, NH_3_, VOCs, 1–3 nm nanocluster aerosols, 1–10 μm total and fluorescent particles, as well as bacterial and fungal microbiota. Emissions from groups of big and small dogs were contrasted, and then compared to those from humans. The findings establish a foundation for a comprehensive understanding of the role of dogs in shaping indoor air quality and their potential contribution to human health through this pathway.

## Materials and Methods

### Climate Chamber

We conducted controlled experiments in a 62 m^3^ climate chamber at the École Polytechnique Fédérale de Lausanne (EPFL) (Figure S1). The chamber was ventilated at 1.44 ± 0.01 h^–1^ air change rate using 100% outdoor air, which was filtered through a newly installed F9 particle filter and then a HEPA filter and an activated carbon molecular filter. Filtered air was introduced through a ceiling supply diffuser and removed through a ceiling exhaust outlet. The chamber’s Heating, Ventilation, and Air Conditioning (HVAC) system maintained the air temperature at 24 ± 0.5 °C and relative humidity at 50 ± 5%. The chamber was sparsely furnished, with a table and chair available for the supervising dog owner. To ensure efficient air mixing, two pedestal fans were placed in opposite corners of the chamber, directed toward the walls. Chamber surfaces, including furniture, were thoroughly cleaned by ethanol wipes and then a steam cleaner with distilled water before the experiments and at regular intervals during the study.

### Experimental Design and Procedure

We recruited two groups of dogs categorized by their sizes for the study: a small dog group consisting of four Chihuahuas and a big dog group including a Tibetan Mastiff, a Newfoundland, and a Mastiff (details in Table S1 and Figure S2). The research protocol was ethically approved by the Canton of Fribourg, Switzerland (Approval No. 34869-2022-18-FR). To meet ethical requirements and facilitate management, each group of dogs participated in the experiments with their common owner, ensuring that only one owner was present in the chamber at any given time. Although we were not able to strictly control dogs’ activities prior to experiments, owners were instructed to maintain a consistent daily routine for their dogs during the experiment week, with no bathing allowed. Additionally, owners were required to follow strict protocols to minimize variability in their own emissions. The evening before each experiment, they showered using provided unscented soap and shampoo. On experimental days, 30 min prior to entering the chamber, they changed into short-sleeved T-shirts and shorts supplied by the researchers. These clothes were prewashed with unscented laundry detergent after purchase, tumble-dried, and individually sealed in zip-lock bags. Only personal phones and water bottles were allowed inside the chamber.

To assess the potential impact of ozone on dog emissions of VOCs and nanocluster aerosols, each group of dogs underwent two chamber sessions per experimental day. In the morning session (ozone level ∼2 ppb), the dogs and their owner remained in the chamber for 2 h without intentional ozone injection. In the afternoon session, an ozone generator (Jelight 600 UV, Jelight Co. Inc.) was activated 10 min after chamber entry, raising and stabilizing the ozone concentration at ∼28 ppb. During each session, the owner sat on a chair and encouraged the dogs to remain calm. Every 30 min, the owner engaged in 15 min of interaction with the dogs, simulating typical daily behavior, before returning to a calm state. Thus, each 2-h session included two engagement periods. During engagements, the owner was instructed to walk together with each dog sequentially for approximately 3–4 min, followed by ∼3 min petting in total. We installed a video camera inside the chamber to monitor (but not record) the dogs’ activities and their interactions with owners. To isolate emissions from owners, we also conducted control tests in which only the owner was present in the chamber, under both ozone conditions and following the same engagement protocols (1 h without ozone, followed by 1 h with ozone; Figure S3). The complete experimental schedule is detailed in Table S2.

### Instrumentation and Data Analysis

We measured CO_2_, NH_3_, VOCs, nanocluster aerosols, 1–10 μm total and fluorescent particles, and bacterial and fungal concentrations and compositions within the climate chamber. The CO_2_ was recorded at 0.5 s intervals using a high-accuracy gas analyzer (LI-850, LI-COR Biosciences GmbH, Germany; range: 0–20,000 ppm, accuracy: ±1.5%). Chamber air was drawn into the analyzer at a rate of 0.75 L/min using an air pump (AirCheck TOUCH, SKC Inc., UK). NH_3_ levels were monitored by a high-precision NH_3_ analyzer at 30-s intervals (LSE NH_3_-1700 Analyzer, LSE Monitors, Netherlands; range: 0–15 ppm, noise: 1 ppb, precision: 2 ppb), with a short 10 cm sampling line to minimize NH_3_ loss.

A Vocus Proton Transfer Reaction Time-of-Flight Mass Spectrometer (Vocus PTR-ToF-MS, Tofwerk AG and Aerodyne Research, Inc.) sampled VOCs through a 0.65 m 14 -inch perfluoroalkoxy (PFA) tubing at ∼100 cm^3^/min from the main air line (1/2-in. PFA) after a PTFE filter to prevent clogging in the capillary. The main air stream was maintained at 12.5 L/min by an external pump, from either the chamber or the supply air.

Nanocluster aerosols (1–3 nm) were monitored using a Nano Condensation Nucleus Counter (Airmodus A11 nCNC System, Airmodus, Finland) at a flow rate of 2.5 L/min. Total and fluorescent particles in the size range 1–10 μm were measured with the Wideband Integrated Bioaerosol Sensor (WIBS NEO, Droplet Measurement Technologies, US) at a sampling rate of 0.3 L/min. Because the instruments for particle sampling were positioned immediately outside the chamber, we sampled the particles with isokinetic core sampling probes at a carrier flow of 5 L/min so as to minimize the sampling loss, and the particle loss was corrected accordingly.^20,23,24^

For microbial sampling, we used eight Personal Environmental Monitor (PEM) samplers distributed throughout the chamber (761-200A, SKC Inc.) that collected airborne PM_10_ particles via inertial impaction onto a 0.8 μm pore size, 37 mm polycarbonate filter membrane. Each sampler operated at 4 L/min during the full 4-h occupied period, including both the morning and afternoon sessions in each experimental day with dogs. For the 2-h experiments with each dog owner, the sampling duration was 2 h to differentiate the emissions from the two dog owners (Table S2). The membranes were subsequently sent to the molecular microbiology laboratory at Finnish Institute for Health and Welfare for quantitative polymerase chain reaction (qPCR) and Next-Generation Sequencing (NGS) analysis to determine both the concentration and composition of bacteria and fungi. To ensure adequate biomass, we combined eight collected filters as one sample for analysis. Details of the analyzing procedures can be found in Section S1. In addition, detailed information on the used real-time instrumentation and microbial analysis methods can also be found in our previous publications.^13,14,20,25–27^

All the instruments were calibrated before the experimental campaign. As shown in Table S2, each experiment had one replicate.

We calculated per-dog emission rates of air pollutants using the material-balance principle, as described in Section S2. We can obtain the total emission rates from dogs and their owner together and from owners only. Their differences represent the contribution of dog emissions and they were further divided by dog number to have emissions per dog.

For CO_2_, NH_3_, and 1–10 μm particles—whose emissions are assumed to be independent from low-level ozone (28 ppb in our experiments),^13,14^ —we calculated emission rates for each session, yielding four data points (two morning and two afternoon sessions) to obtain their average ± std values per group. Additionally, time series of these three were averaged into 5 min blocks to avoid autocorrelation and then used for nonparametric Mann–Whitney U tests to evaluate the significance of the differences between owner-only and dogs + owner conditions. In contrast, because VOCs and nanocluster aerosols are sensitive to ozone’s presence,^17,18^ we segregated ozone-absence and ozone-present sessions, resulting in two data points per dog group under each ozone condition, for which only average emission rates could be reported. For VOC emissions specifically, we ensured data robustness by focusing on species that met three criteria: (1) The concentration difference between the occupied and empty chamber exceeded 3× the compound’s limit of detection (LOD); (2) During the occupied period, the concentration difference between experiments with and without dogs exceeded 3× LOD, and the relative difference in overall emission rates between dogs and owners was >25%; and (3) The compound signals exhibited good reproducibility across replicate sessions (difference <25%). For microbial emissions, since morning and afternoon sessions used the same samplers, we obtained two data points per dog group and reported average values only. In addition, we calculated Chao1 and Shannon indices to quantify microbial diversity.

## Results and Discussion

### CO_2_ and NH_3_ Emissions

Figure 1a,b illustrate a clear rise in indoor CO_2_ and NH_3_ concentrations immediately after dogs and their owner entered the environmental chamber (time = 0 h). Upon their departure (time = 2 h), there were sharp decreases in the concentrations. During engagement activities, including owner-only interactions, CO_2_ and NH_3_ levels also rose significantly, showing that movement and activity increased emission intensities for both dogs and humans. Notably, NH_3_ concentrations lagged slightly behind CO_2_, likely due to NH_3_’s much greater affinity to surfaces.^13^ The differences in concentration curves when dogs and humans were present together versus only humans show that dogs are potent sources of CO_2_ and NH_3_. CO_2_ showed significantly higher concentrations during dogs + owner sessions compared with owner-only conditions for both small dogs (*p* = 10^–4^) and big dogs (*p* = 10^–8^). Similarly, NH_3_ levels were also considerably higher when dogs were present in the chamber (*p* = 10^–4^ and 10^–11^ for small and big dogs, respectively).

**Figure 1:**
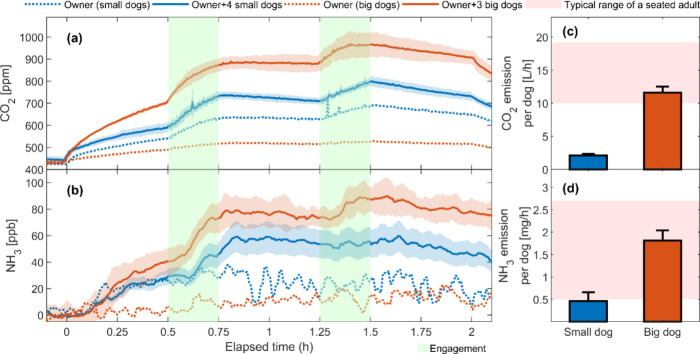
Time series of CO_2_ and NH_3_ concentrations (a, b) and their emission rates in comparison to the typical range of a seated adult (c, d). In (a, b), solid lines represent the average values across four experimental sessions per dog group, with shaded areas representing standard deviations. Dashed lines represent results from the sole owner’s experiment. Green shaded areas represent periods of (mimicked) dog engagement. In (c, d), bars represent the average emission rates per dog across the four experimental sessions per dog group, with error bars representing standard deviations. The red shaded areas represent typical CO_2_ and NH_3_ emission ranges for a seated adult.

Figure 1c,d show emission rates for CO_2_ and NH_3_ per dog, alongside the range of sedentary human emission rates. A big dog emitted an average of 1.8 mg/h NH_3_, more than three times the amount of a small dog (0.5 mg/h), both of which were within the typical range for a seated adult (0.5–2.7 mg/h).^12,13^ Similarly, the average CO_2_ emission rate of a big dog (12 L/h) was comparable to those of a seated adult (10–19 L/h)^9,13,28,29^ and was six times higher than the average emission rate of a small dog (2 L/h).

To quantify body size, we introduced a “dog body mass index” (DBMI), analogous to human BMI, calculated as weight divided by the square of height (details in Table S1). As expected, higher DBMI values were associated with increased CO_2_ and NH_3_ emission rates (Figure S4), suggesting these emissions primarily originate from endogenous metabolic processes. Similar to humans, we presume that dogs’ CO_2_ emissions are primarily respiration-driven, while NH_3_ emissions stem from protein metabolism.^30,31^

Notably, the NH_3_-to-CO_2_ emission ratio was higher for dogs than for humans, potentially reflecting differences in diet, metabolic pathways, and breathing patterns. While total respiratory volumes are similar between humans and dogs,^32^ dogs typically exhibit higher respiratory rates, especially in small breeds.^33^ Under heat stress (e.g., elevated ambient temperature or physical activity), dogs may reach respiratory rates of up to 300 breaths per min, due to their limited ability to dissipate heat via evaporation, in contrast to humans.^32^

### VOC Emissions

Figure 2 illustrates the types and intensities of VOC emissions from dogs, as well as the influence of dog size and ozone presence on these emissions. Without intentional ozone injection (ozone level inside the chamber: 2 ppb), we were able to discern four VOCs that were potentially significantly emitted by the small dogs, with C_5_H_11_O^+^ (likely pentanal) being emitted at an average rate higher than that of the dog owner, reaching 45 μg/h/dog. The big dogs emitted more than ten VOCs, with five of these VOCs emitted at an average rate exceeding that of the owner, including acetone, 6MHO, and nonanal (with dog/owner emission ratios of 1.1, 1.4, and 1.4, respectively). In particular, the average emission rate of a compound corresponding to mass to charge ratio of 103.0753 assigned to C_5_H_11_O_2_^+^ from the big dogs reached approximately 60 μg/h/dog, 15 times that of its owner (red circle in Figure 2). Its exact chemical structure could not be determined due to instrument limitations. Potential compounds for this signal could be valeric acid or isovaleric acid, which has been associated with dogs’ feces owing to dietary and gut-microbiome metabolites.^34^ In addition, dog skin lipids consist of wax diesters, sterol esters, and branched fatty acids,^35,36^ whose oxidation can generate C_5_ acyl fragments. Given these multiple plausible pathways and the potential for cofragmentation in instrument analyses,^37^ the chemical identity of C_5_H_11_O_2_^+^ remains unresolved, and its absolute emission rate should be interpreted with caution (Table S3). Based on current findings, we cannot conclusively determine whether these VOCs are endogenous or exogenous. For instance, acetone is likely endogenously produced through physiological processes, as found in dogs’ exhaled air.^38^ In contrast, 6MHO, typically known as a marker of ozone-human skin lipid reactions,^19^ is likely transported into the chamber on the dogs’ fur. Similarly, nonanal may also originate exogenously from bathing agents^39^ or human petting.^19,40^

**Figure 2:**
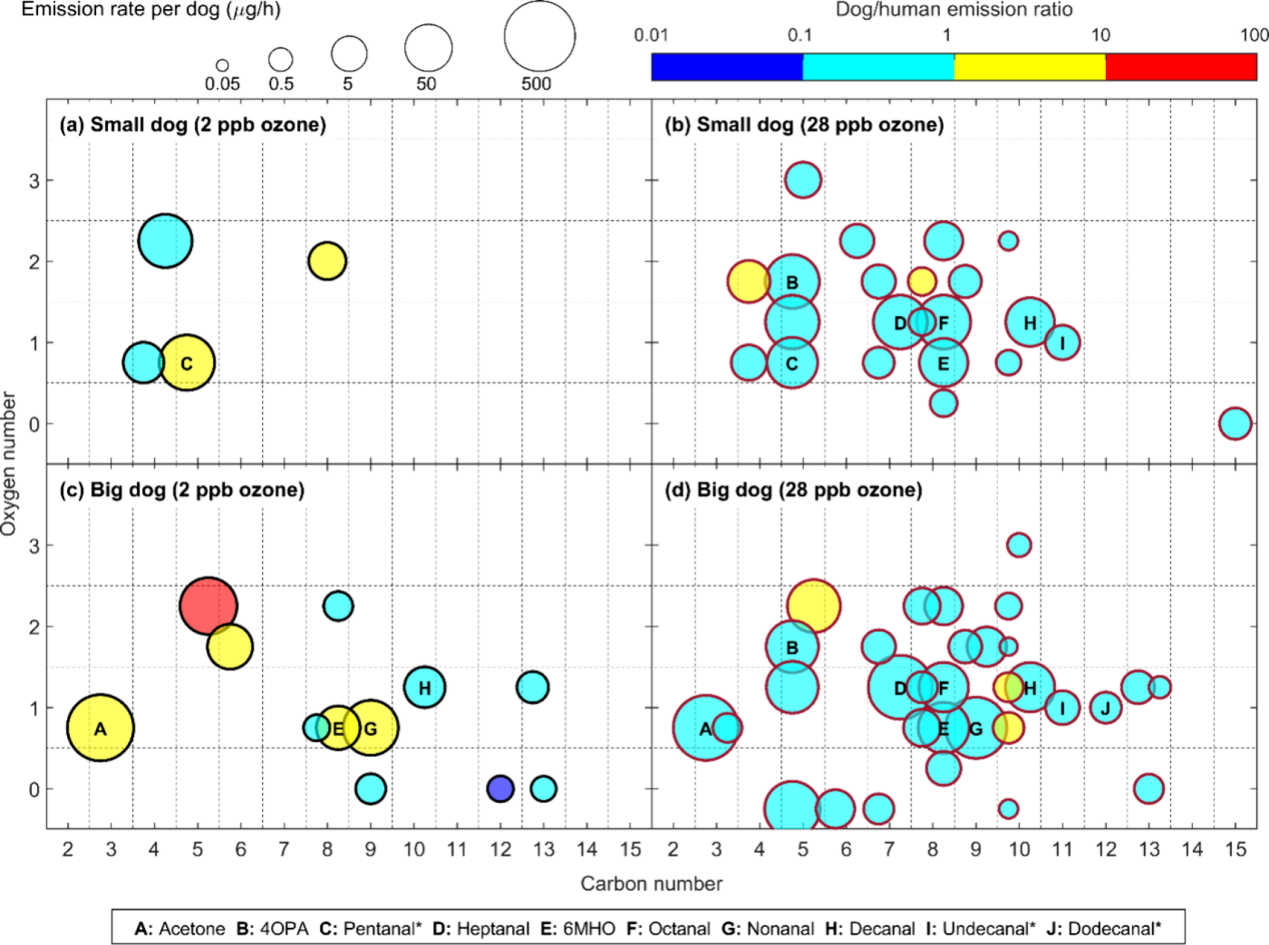
Emission rates of main VOCs emitted by dogs (per dog) and their dependence on dog size and ozone level. The size of the bubbles represents the emission rate, and the color represents their ratio to human emissions. Results for dogs are average emission rates across each experiment and its replicate. Notably, compounds C (C_5_H_11_O^+^), I (C_11_H_23_O^+^), and J (C_12_H_25_O^+^) were identified as pentanal, undecanal, and dodecanal, respectively, with medium confidence, whereas other compounds were identified with high confidence (such as 4OPA and 6MHO, which have been previously reported as ozone-human reaction products).

In contrast, during the ozone experiment (28 ppb), both groups of dogs exhibited increased overall emission rates resulting in a larger VOC diversity, especially due to oxygenated VOCs within the C_5_–C_11_ range (Table S3). This suggests that chemical reactions between ozone and dog skin and fur surfaces generated gaseous products. When focusing on the primary VOC products, many were similar to those produced by ozone-human reactions. Human skin oils are approximately 12% squalene, followed by unsaturated fatty acids and esters,^41–44^ all of which can quickly react with ozone to produce various aldehydes and ketones.^19^ For instance, 4OPA and 6MHO are typical products of ozone reacting with squalene in human skin oils, while aldehydes like heptanal, octanal, and decanal result from the ozonolysis of unsaturated fatty acids in human skin.

It is noteworthy, however, that dog skin oil composition differs significantly from that of humans, being primarily composed of cholesterol esters, ceramides, and wax esters, with low levels of unsaturated fatty acids and no squalene.^35,36^ Furthermore, ozone readily decomposes on surfaces and thus is likely to be depleted strongly when mixing through the fur. Consequently, the ozonation rate of dog skin oils should be considerably lower than that of human skin and is unlikely to directly produce squalene-derived ozonolysis products like 6MHO and 4OPA. The VOC emission rates of dogs during the ozone experiment were notably lower than those of their owners. We hypothesize that human skin oils transferred onto the dog’s fur through physical contact through petting and subsequently reacted with ozone in the chamber. We note, however, that this remains a hypothesis consistent with the chemical evidence on ozone-squalene reactions rather than a mechanism directly demonstrated in this study, because petting frequency and skin oil transfer were not quantitatively controlled. Direct confirmation of the mechanism was therefore beyond the scope of this study and should be interpreted as a likely but unconfirmed interpretation of the observed VOC patterns.

Notably, for big dogs, acetone was the primary emitted VOC (∼312 μg/h/dog on average). In the absence of ozone, the emission rate was slightly higher than that of its owner (ratio = 1.1); however, with ozone present, this ratio dropped to 0.6. We speculate that, similar to humans, dogs may exhale acetone as a metabolic byproduct of ketone metabolism,^10,38,45,46^ with emission rates potentially higher than in humans. However, in the presence of ozone, acetone can form as a product of ozone reacting with human skin.^19^ Since this pathway is absent in dogs, their acetone emission rate was lower than that of humans under ozone-present conditions. Additionally, the difference in acetone emissions between ozone-present and ozone-absent conditions remained within 25% for big dogs (274 vs 349 μg/h/dog). This supports the notion that acetone emissions from dogs were not substantially altered by the presence of ozone. Similarly, the aforementioned compound C_5_H_11_O_2_^+^ also showed lower dog-to-human emission ratio in ozone-present experiments (3.5 vs 15.4), indicating that it may also originate from ozone-human skin chemistry.

It should be noted that, for all tentatively identified VOCs that lack calibration standards, their absolute emission rates remain uncertain (Table S3). These values should be interpreted cautiously given the uncertainties in fragmentation patterns, isomeric contributions, and sensitivity factors. We therefore emphasize relative differences—particularly dog-to-human ratios. Because all species were measured with the same instrument settings, any calibration biases or fragmentation uncertainties are expected to affect the dog and human signals proportionally. Thus, while the absolute magnitudes carry uncertainty, the relative differences between dogs and humans are reliable.

### Particle and Microbial Emissions

Dogs did not exhibit considerable emissions of 1–3 nm nanocluster aerosols (NCAs), even when ozone was present (Figure S5). This is likely because NCAs primarily form through chemical reactions between ozone and squalene^17,20,47^ in human skin—a compound absent in dogs. When both dogs and humans were present in the ozone-rich chamber, the slightly higher NCA concentrations, relative to the human-alone condition, may have resulted from ozone reacting with squalene transferred from human skin to dog fur, as previously discussed.

In contrast, both small and big dogs were substantial sources of 1–10 μm particulate matter, with emission rates per dog surpassing the average of their owners (Figure 3). When dogs and humans entered the chamber, total and fluorescent particle levels rose markedly, with distinct peaks observed during engagement periods (Figure S6). The concentration differences between “dog + owner” and owner-only sessions were significant (*p* = 10^–12^ and 10^–5^ for small and big dogs, respectively) This indicates that dogs are important sources of indoor particulate matter. A small dog emitted on average 0.61 mg/h of 1–10 μm total particles, exceeding the emissions from both a big dog (0.42 mg/h) and the owner (0.39 mg/h), despite having a considerably smaller body size and surface area. This may reflect higher activity levels observed in small dogs during the experiments, which initiated more particle emissions and resuspensions.

**Figure 3:**
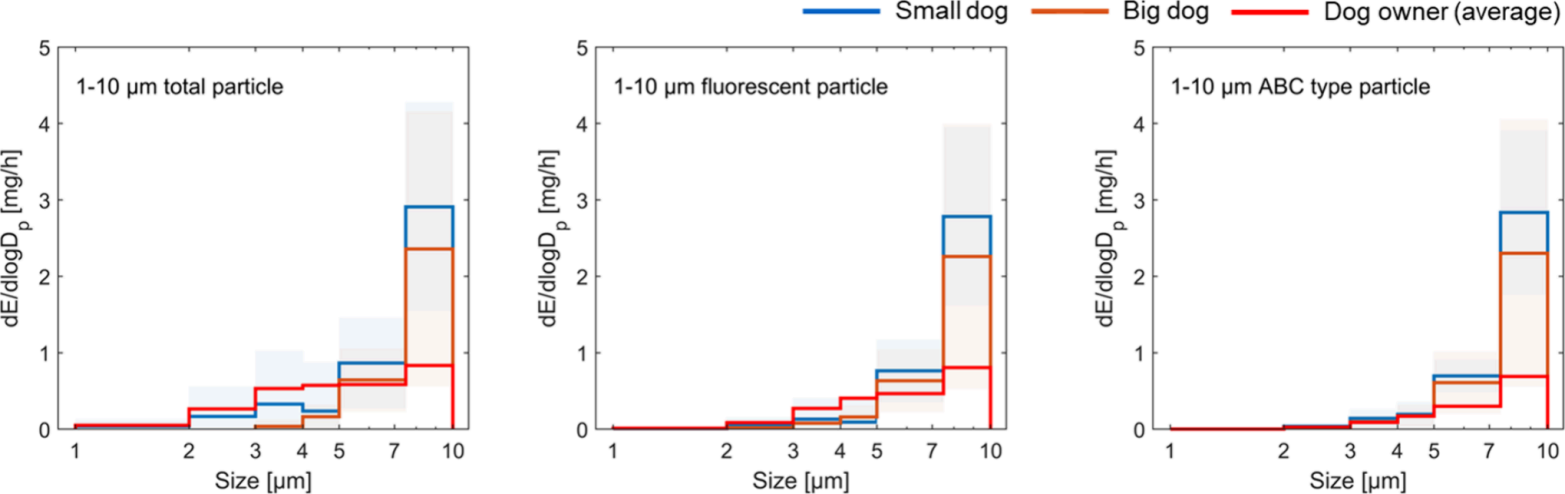
Size-resolved emission rates per dog of 1–10 μm total, fluorescent, and the dominant ABC-type particles. For dogs, solid lines represent the average values across four experimental sessions for each dog group, with shaded areas representing standard deviations. For dog owners, solid lines represent the average results from the individuals measured separately. ABC-type particles are those that trigger fluorescence in all three WIBS channels (A, B, and C), typically corresponding to strongly fluorescent particles.

Analysis of particle size distribution revealed that, compared to humans, dogs emitted fewer particles in the 2–5 μm range (15 and 5% for a small and a big dog, respectively vs 43% for the owner), but a greater proportion in the 5–10 μm range (83 and 95% for a small and a big dog, respectively vs 53% for the owner). These differences likely reflect contrasting emission sources: humans wore clean clothing, so their particles likely originated from skin shedding and clothing fiber fragments.^14,48^ In contrast, dog-emitted supermicron particles likely stem from their own skin and fur, as well as from external particles adhered to their fur, which acts as a vector for outdoor-to-indoor particle transport. The dominance of 5–10 μm particles in dog emissions has important exposure implications owing to disproportionately higher mass emissions compared with human-derived particles. Although such coarse particles settle rapidly under quiescent conditions, they can be released through resuspension from floors, fur, and surfaces. This is consistent with the pronounced activity-related concentration spikes observed during engagement periods (Figure S6), suggesting that dog-derived coarse particles may intermittently dominate the airborne particle mass burden in humans’ breathing zone. In addition to inhalation, the mass-rich deposition of these particles onto indoor surfaces may enhance opportunities for indirect exposure through contact and fomites.

A large majority of the emitted 1–10 μm particles were fluorescent: 84% for small dogs and 98% for big dogs. These fluorescent particles were predominantly of the ABC type, especially among the larger size fractions. ABC-type particles are those that trigger fluorescence in all three WIBS channels (A, B, and C), typically corresponding to strongly fluorescent particles.^14,49^ While fluorescent particles are often regarded as proxies for biological material, it is important to note that not all detected fluorescent particles are biologically active, as abiotic particles can also exhibit fluorescence due to certain surface compounds or coatings.^14,50^

QPCR results revealed quantitative differences in bacterial and fungal emissions between small and big dogs (Figure 4a–c). Big dogs emitted Gram-negative bacteria on average at two times the rate of the owners on average (2.0 vs 1.0 million cells/h per dog or person), while the small dog showed negligible Gram-negative bacterial emissions. Similarly, for Gram-positive bacteria, the big dogs’ emission rate (0.6 million cells/h/dog) was higher than that of both the small dogs and humans. Big dogs also emitted fungi at a higher rate than the small dogs (0.5 vs 0.2 million cells/h/dog), with both dogs’ fungal emissions exceeding those of their owners (on average 0.1 million cells/h/person).

**Figure 4:**
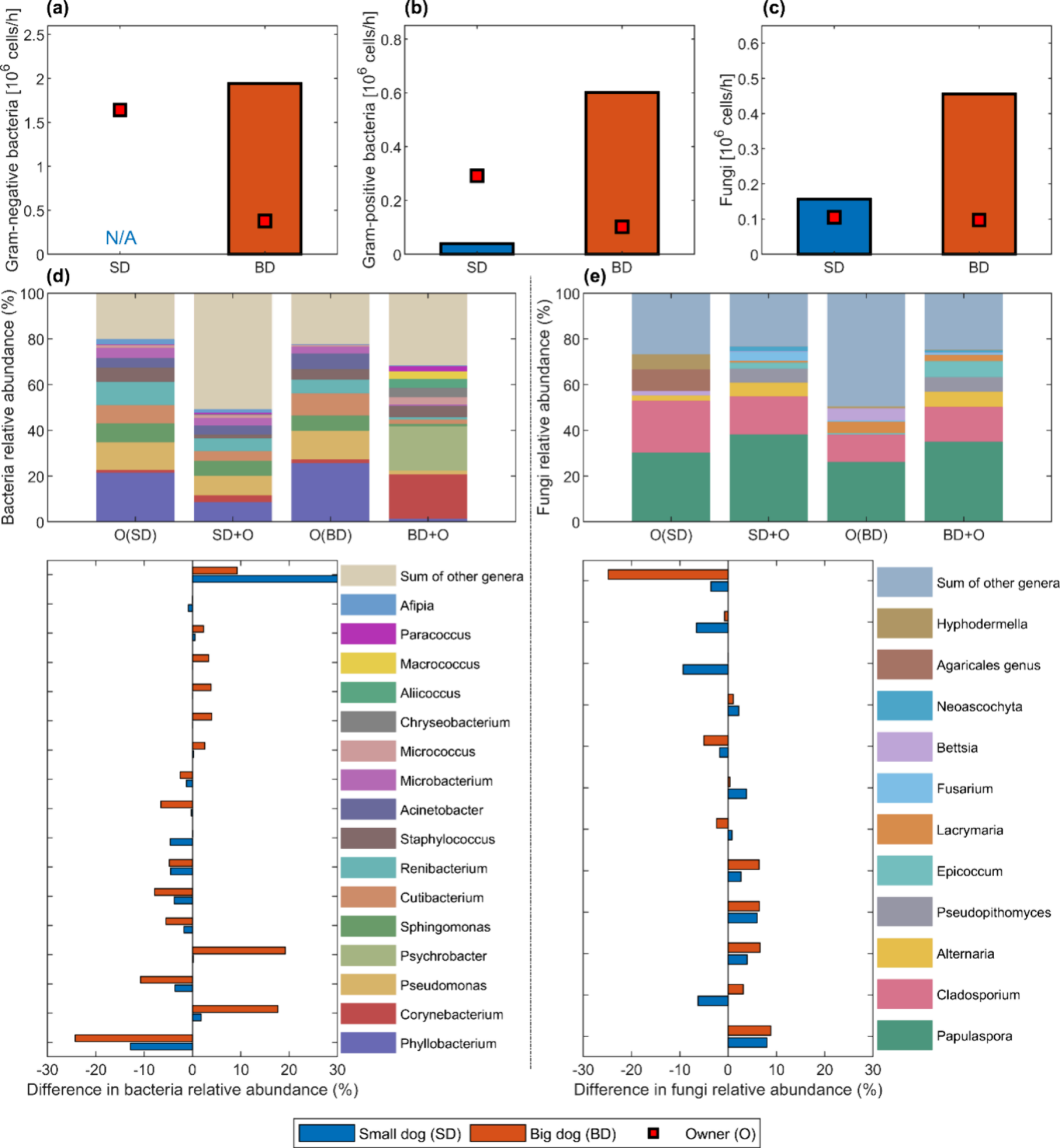
Microbial emission rates per dog based on qPCR results (a–c) and comparing compositions of the most abundant detected bacterial (d) and fungal (e) taxa based on amplicon sequencing between dogs + owner and owner-alone experiments. Results for dogs are average values between each experiment and its replicate. The “N/A” for emissions of Gram-negative bacteria from a small dog (a) was owing to the lower indoor average Gram-negative bacteria level in experiments with small dogs and their owner relative to that with the owner alone. The lower plots in (d) and (e) present differences in bacteria/fungi relative abundances between dogs + owner and owner-alone experiments (positive values indicate contributions from dog emissions to the microbial composition in the chamber air).

When assessing the microbiota composition of the chamber air and focusing on the most abundant bacterial genera (defined as taxa with a mean relative abundance of >1% across samples), we observed that both dog owners’ emissions were similarly dominated by bacterial taxa sourced from human microbiota and the environment, including *Phyllobacterium*, *Pseudomonas*, *Sphingomonas*, *Cutibacterium*, *Renibacterium*, *Staphyloccus*, *Acinetobacter*,and *Microbacterium* (Figure 4d). However, with the introduction of different dog groups into the chamber, the airborne bacterial communities shifted considerably, leading to an increased taxa richness and diversity (Figure 5). This finding aligns with literature reporting that dogs can alter household microbial composition.^51–53^ Yet, the changes induced by the big and small dogs followed different patterns. For the big dogs, there was a marked increase in the relative abundances of *Corynebacterium* and *Psychrobacter* in the chamber air, with some increase also observed in *Aliicoccus* and *Macrococcus* (Figure 4d). These taxa align well with known skin bacterial taxa of healthy dogs.^54,55^ For the small dogs, no major changes were observed in the top abundant taxa and the most considerable change was an increase in the proportion of sequences derived from other, less abundant genera (“sum of other genera”).

**Figure 5:**
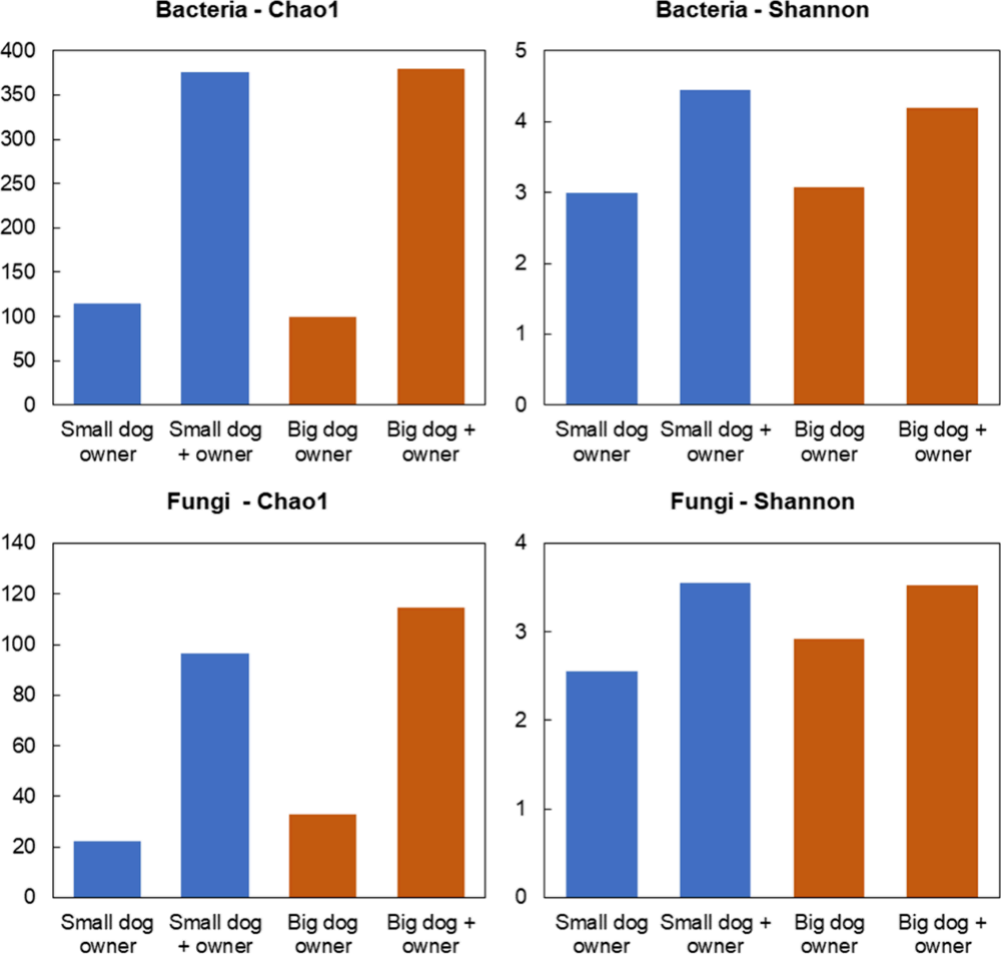
Chao1 taxa richness and Shannon diversity indices of bacteria and fungi emissions from dogs and their owners. *y*-Axes in Chao1 plots present predicted number of ASVs and the Shannon diversity metric in the Shannon plots. Results for dogs are average values between each experiment and its replicate.

The fungal profiles of the two dog owners differed noticeably and were largely dominated by environmental taxa, yet this disparity almost completely disappeared when the dogs entered the chamber (Figure 4e). Specifically, the big dog owner introduced a greater proportion of sequences from the fungal genera, *Bettsia* and *Lacrymaria*, while the small dog owner’s emissions showed a larger proportion of *Cladosporium*, an *Agaricales* genus, and *Hyphodermella*. Both owners had a similarly strong contribution from *Papulaspora*. Once the dogs entered the chamber, however, the fungal composition became more consistent but also more diverse (Figure 5), with *Papulaspora*, *Cladosporium*, *Alternaria*, *Pseudopithomyces*, and *Epicoccum* (more in big dogs) predominating. Considering these taxa being rather environmental than associated with dogs’ mycobiome, these dominant fungal types may have adhered to the dogs during environmental contacts prior to the experiments and were subsequently released in the chamber. Given that both groups of dogs roamed outside the experimental area prior to the experiments, they may also have accumulated similar fungal spores from the same area outside the chamber, leading to the comparable fungal emission profiles observed.

Dogs significantly altered the air microbiota in the chamber, leading to increased bacterial and fungal loads, elevated microbial richness and diversity, and a higher proportion of environmentally derived taxa. These changes are consistent with prior hypotheses that pet ownership may influence indoor microbial exposure in ways that affect human immune development and respiratory health. For instance, *Corynebacterium* is part of the normal human and canine skin microbiota but can influence skin chemical ecology through lipid metabolism, including the production of short-chain fatty acids that modulate skin pH and barrier function.^56^ Such metabolites have been suggested to shape host immune responses, potentially contributing to the heterogeneous associations reported between dog exposure and allergic or respiratory outcomes.^57^ *Psychrobacter*, a psychrotolerant environmental genus, has been associated with outdoor bioaerosol sources^58,59^ and may reflect the dog’s role as a mechanical vector transporting environmental microorganisms indoors. While these genera are not considered primary pathogens in healthy individuals, their introduction may modify the overall microbial diversity and functional metabolic potential of indoor air. However, the health implications of such exposures remain unclear as existing evidence on the relationship between dog-associated indoor microbiota and allergic or respiratory outcomes is mixed and sometimes contradictory.^3–8,60^ In studies on associations between indoor microbial exposure and allergic health outcomes, there is little to no consensus on specific, nonpathogenic microbial taxa that would be consistently and strongly linked to either protective or adverse effects.^60^ Therefore, while our findings demonstrate that dogs are significant sources and modifiers of indoor microbial communities, it remains premature to infer the health relevance of the bacterial and fungal genera identified in our analyses.

### Limitations

Due to ethical considerations and the need for effective animal handling, dogs were only allowed to enter the chamber alongside their owners. This copresence introduced uncertainty in attributing emission rates specifically to the dogs, as it required the assumption that the owner’s emissions remained independent of the dogs’ presence. Additionally, although we regulated the owners’ diet, clothing, and hygiene practices during the experimental period to minimize variability, human chemical emissions can fluctuate over time.^18^ As a result, subtracting owner-only control data may have introduced some error in estimating dog-specific emission rates. However, most pollutant concentrations, particularly in sessions involving large dogs, showed significant increases or changes relative to owner-only sessions, reducing concerns over this potential source of error. Moreover, each experiment involved groups of 3–4 dogs, meaning that even modest dog-human emission contrasts produced amplified concentration signals at the chamber scale.

Similarly, ethical and logistical constraints prevented strict control over dogs’ diet, hygiene, and environmental exposures during the experiment week, introducing possible variability in emissions, especially for exogenous pollutants. For the same reasons, we did not perform direct sampling from the dogs, such as swab-based microbiota analyses, which could have strengthened source attribution of pollutants detected in the chamber air.

Due to time and resource constraints, each dog experiment had only one replicate. While this limited our ability to perform statistical testing for VOCs and microbial emissions, we prioritized pollutants that were reliably detected. Specifically for VOCs, we included only those compounds for which concentration differences between each experiment and its replicate remained within 25%. Additionally, our prior studies^13,17,18,20,61,62^ have demonstrated the repeatability of the experimental protocols used here, lending support to the robustness of the observed trends. Regarding emission rate calculation, although concentrations did not always reach steady state within the 2-h period, emission rates were computed using the full transient mass-balance method (Section S2), consistent with our previous validated chamber studies.^13,14^ While some uncertainty is inherent to transient conditions (within 13% for NH_3_, detailed in Section S2 and Yang et al.^13^), this method has been shown to provide robust emission estimates.

Another limitation is that we could not distinguish between endogenous and exogenous pollutant emission mechanisms, particularly for pollutants influenced by prior outdoor exposure. Finally, instrumentation limitations prevented us from confirming the precise structure and composition of all VOCs emitted by the dogs. Some signals presented in Figure 2 may represent a mixture of isomeric compounds sharing the same mass to charge ratio rather than complete compounds. In addition, emissions especially for aldehydes can be seen as a lower limit due to fragmentation. Consequently, the emission rates reported in Table S3 should be interpreted with caution, especially for compounds without calibration. Nevertheless, the ratios of dog-to-human emissions were less influenced and remained solid in general.

### Implications and Future Outlook

Despite dogs having accompanied humans for thousands of years, especially now as household pets, we still know surprisingly little about how they affect indoor air quality and human physiological health. This study provides the first controlled experimental evidence and quantitative assessment showing that dogs are potent indoor emitters of a wide range of gaseous pollutants, particulate matter, and microorganisms. These findings offer a new perspective on indoor environmental exposures, emphasizing the need to consider pets —particularly dogs — as significant and dynamic contributors to indoor air composition with potential health relevance.

Our results indicate that, much like humans, dogs emit pollutants through both endogenous (e.g., metabolic processes) and exogenous pathways (e.g., surface-mediated transport). Endogenous emissions were influenced by factors like body size, activity level and presumably diet. Exogenous emissions suggest that dogs act as mobile vectors, transporting chemicals, particulate matter, and microbes from outdoor or other environments into indoor spaces—an effect likely amplified in larger or longer-haired dogs. This underscores the need to better understand the role of dogs in cross-environmental contamination and its potential health effects.

Notably, current indoor air quality management strategies rarely account for pets as emission sources. Our findings challenge this oversight, showing that dogs can emit pollutants such as CO_2_, NH_3_, VOCs, and supermicron particles at rates comparable to or exceeding those of a human adult, depending on their size. This suggests that indoor air quality standards, building ventilation design, and broader environmental control strategies should explicitly account for pets as active emission sources within occupied spaces. In addition, because inlet ozone concentrations were not continuously measured, we were unable to estimate a dog-specific ozone deposition velocity; determining whether and to what extent dogs act as indoor ozone sinks will require a dedicated experimental design and remains an important direction for future work.

Looking ahead, future studies could expand on these findings by mapping the full spectrum of pollutants emitted by dogs and examining the health impacts of specific dog-associated pollutants on both humans and animals themselves. While our study grouped dogs by body size to ensure sufficient signal strength, it did not control for breed-specific traits, physiological condition, hygiene, diet, or underlying health status—all of which may influence emission profiles. Future research could focus on breed-level variation and incorporate a broader range of endogenous factors to gain a more nuanced understanding of emission mechanisms. Additionally, extending this research to other household pets, such as cats, rabbits, or rodents, could further enrich our knowledge of animal-related indoor exposures.

In summary, this study highlights the important yet often-overlooked role of dogs in shaping indoor air quality. As pet ownership continues to rise globally, integrating pets into indoor air quality research, exposure modeling, and environmental control strategies will be crucial for designing healthier indoor environments—not just for humans, but for the animals that live alongside them.

## Supplementary Material



## Data Availability

All data are available in the main text or the Supporting Information.

## Notes

Details of microbial analysis methods; calculation of air pollutant emission rates; schematic layout of the chamber setup; four small dogs (left) and three big dogs (right) inside the chamber during experiments; experiment procedure for owner only (upper, D0) and dogs and owner together (lower, D1–D4); relationship between the DBMI and dogs’ (a) CO_2_ and (b) NH_3_ emission rates; 1–3 nm nanocluster aerosol emissions from dogs and their owners; an example of 1–10 μm total and fluorescent particle concentrations during the morning and afternoon session; detailed information about the two dog groups; experimental schedule; and emission rates of main VOCs emitted from dogs and comparison with their owners (PDF)
