# Supplementary material for: Our Best Friends: How Dogs Alter Indoor Air Quality
Source: Environ Sci Technol. 2026 Feb 2;60(8):6404–14. doi: 10.1021/acs.est.5c13324 (PMC12961941; doi:10.1021/acs.est.5c13324)
Supplement: Supplementary file 1 [file es5c13324_si_001.pdf]

Supplementary Materials for

Our best friends: How dogs alter indoor air quality

Authors

Shen Yang,[1,2] Nijing Wang,[3] Tatjana Arnoldi-Meadows,[3] Gabriel Bekö,[4] Meixia Zhang,[1] Marouane Merizak,[1] Pawel Wargocki,[4] Jonathan Williams,[3] Martin Täubel,[5,6] Dusan Licina[1*]

Affiliations

[1]Human-Oriented Built Environment Lab, School of Architecture, Civil and Environmental Engineering, École Polytechnique Fédérale de Lausanne (EPFL), 1015 Lausanne, Switzerland

[2]School of Architecture, Southeast University, 210096 Nanjing, China

[3]Max Planck Institute for Chemistry, Hahn-Meitner Weg 1, 55128 Mainz, Germany

[4]International Centre for Indoor Environment and Energy, Department of Environmental and Resource Engineering, Technical University of Denmark, 2800 Kongens Lyngby, Denmark

[5]Lifestyles and Living Environments, Department Public Health, Finnish Institute for Health and Welfare, P.O. Box 95, 70701 Kuopio, Finland

[6]Department of Civil Engineering, School of Engineering, Aalto University, P.O. Box 12100, 00076 Espoo, Finland

Corresponding author:

Dusan Licina (dusan.licina@epfl.ch)

This PDF file includes:

Supplementary Text
Figs. S1 to S6
Tables S1 to S3
References (1 to 12)

Supplementary Text

Section S1. Details of microbial analysis methods

From each experiment we created three chamber air samples by combining three or two filters in the DNA extractions into one sample, with the goal to increase microbial material in the air samples. DNA extraction was performed using ZymoBIOMICS DNA Miniprep kit (Zymo Research, USA) involving both chemical and mechanical lysis of microbial cells via bead beating and including salmon testes DNA as internal standard (Sigma-Aldrich Co., USA) [1]. QPCR analysis was done using previously published assays targeting total fungal DNA, Gram-positive and Gram-negative bacteria [2]. QPCR reactions were performed as described earlier. Relative quantification method was used to calculate numbers of cell equivalents (CE) in each qPCR reaction [3]. The combined sampled air volumes were calculated from the individual PEMs and results from the quantitative PCR analysis were reported as cell equivalents per m^3 of chamber air. Bacterial and fungal amplicon sequencing targeting the bacterial 16S rRNA gene (primers 341f/785r)[4] and fungal internal transcribed spacer region 1 (primers ITS1f/ITS2r)[5] was done at the commercial sequencing service provider LGC Genomics (Berlin, Germany) on a MiSeq v3 platform producing paired end reads of 300bp each. Details on PCR conditions and sequencing have been described earlier [6]. Amplicon sequence variants (ASVs) were formed using the dada2 package, version 1.18.0 [7]. Taxonomy was assigned to ASVs using the SILVA taxonomic training data formatted for dada2 (Silva version 138)[8] and the UNITE general FASTA release, respectively [9]. Threshold method in decontam package [10] was used for sample decontamination against blank samples, applying threshold values of 0.5 for fungi and 0.3 for bacteria.

Section S2. Calculation of air pollutant emission rates

The average emission rate of air pollutants during a given period was calculated based on the material-balance equation:

$$V\frac{\mathrm{d}C}{\mathrm{d}t} = E - (\alpha + \beta)VC + \alpha V C_{out} \quad (1)$$

where V is the chamber volume, 62 m^3; C is the gas concentration measured inside the chamber; α is the air change rate, 1.44 h^{-1}; C_{out} is the outdoor gas concentration, approximated by the 20-min average values before participants entering the chamber in each experiment; β is air-pollutant specific deposition rate, which only applied for particles in our study, obtained by fitting the decay curve after dogs and owners exited the chamber; and E is the total emission rate.

We can have emission rates estimated by discretizing Equation 1, from which we got:

$$E = V\left(\alpha(\bar{C} - C_{out}) + \beta\bar{C} + \frac{C(T)-C(0)}{T}\right) \quad (2)$$

where $\bar{C}$ is the average air pollutant concentration during a given period; $C(T)$ is the air pollutant concentration and the end of the given period (5-point average); $C(0)$ is the air pollutant concentration at the beginning of the given period (5-point average); and T is the duration of the given period. This discretization method has been widely used for emission rate calculation of "sticky" materials, such as airborne particles and NH_3. It can also reduce the uncertainty when assuming a steady state.

It is worth mentioning that NH_3 is known as a sticky gas that can be absorbed on chamber surfaces, including walls, furniture, and human surfaces. This property may introduce bias to gas-phase NH_3 measurement and emission rate calculation using Equation 2 when neglecting the deposition rate. The absorbed amount of NH_3 onto surfaces depends on the gas-phase NH_3 concentration, surface-bounded NH_3, surface properties, and air temperature and humidity [11]. It brings challenges to

calculate the NH_3 deposition rate using the decay-curve-fitting because: (1) owing to the desorption of NH_3 from the chamber surfaces, we often obtained negative deposition rate, meaning that the NH_3 surface removal rate was lower than the air change rate; and (2) due to the build-up of surface-bounded NH_3, the obtained deposition rate from the unoccupied period cannot represent the true absorption of NH_3 by chamber surfaces during the prior occupied period. Nevertheless, our previous estimation using the same chamber settings indicated that the uncertainty caused by the absorption/desorption processes of NH_3 was within 13% [12].

We can obtain the total emission rates from dogs and their owner together and from owners only. Their differences represent the contribution of dog emissions and they were further divided by dog number to have emissions per dog.

References

(1) Haugland, R. A.; Siefring, S. C.; Wymer, L. J.; Brenner, K. P.; Dufour, A. P. Comparison of Enterococcus Measurements in Freshwater at Two Recreational Beaches by Quantitative Polymerase Chain Reaction and Membrane Filter Culture Analysis. *Water Res* **2005**, *39* (4), 559–568. https://doi.org/10.1016/j.watres.2004.11.011.

(2) Kärkkäinen, P. M.; Valkonen, M.; Hyvärinen, A.; Nevalainen, A.; Rintala, H. Determination of Bacterial Load in House Dust Using QPCR, Chemical Markers and Culture. *Journal of Environmental Monitoring* **2010**, *12* (3), 759. https://doi.org/10.1039/b917937b.

(3) Haugland, R. A.; Varma, M.; Wymer, L. J.; Vesper, S. J. Quantitative PCR Analysis of Selected Aspergillus, Penicillium and Paecilomyces Species. *Syst Appl Microbiol* **2004**, *27* (2), 198–210. https://doi.org/10.1078/072320204322881826.

(4) Klindworth, A.; Pruesse, E.; Schweer, T.; Peplies, J.; Quast, C.; Horn, M.; Glöckner, F. O. Evaluation of General 16S Ribosomal RNA Gene PCR Primers for Classical and Next-Generation Sequencing-Based Diversity Studies. *Nucleic Acids Res* **2013**, *41* (1), e1–e1. https://doi.org/10.1093/nar/gks808.

(5) Smith, D. P.; Peay, K. G. Sequence Depth, Not PCR Replication, Improves Ecological Inference from Next Generation DNA Sequencing. *PLoS One* **2014**, *9* (2), e90234. https://doi.org/10.1371/journal.pone.0090234.

(6) Dockx, Y.; Täubel, M.; Bijnens, E. M.; Witters, K.; Valkonen, M.; Jayaprakash, B.; Hogervorst, J.; Nawrot, T. S.; Casas, L. Residential Green Space Can Shape the Indoor Microbial Environment. *Environ Res* **2021**, *201*, 111543. https://doi.org/10.1016/j.envres.2021.111543.

(7) Callahan, B. J.; McMurdie, P. J.; Rosen, M. J.; Han, A. W.; Johnson, A. J. A.; Holmes, S. P. DADA2: High-Resolution Sample Inference from Illumina Amplicon Data. *Nat Methods* **2016**, *13* (7), 581–583. https://doi.org/10.1038/nmeth.3869.

(8) Quast, C.; Pruesse, E.; Yilmaz, P.; Gerken, J.; Schweer, T.; Yarza, P.; Peplies, J.; Glöckner, F. O. The SILVA Ribosomal RNA Gene Database Project: Improved Data Processing and Web-Based Tools. *Nucleic Acids Res* **2012**, *41* (D1), D590–D596. https://doi.org/10.1093/nar/gks1219.

(9) Põlme, S.; Abarenkov, K.; Henrik Nilsson, R.; Lindahl, B. D.; Clemmensen, K. E.; Kauserud, H.; Nguyen, N.; Kjøller, R.; Bates, S. T.; Baldrian, P.; Frøslev, T. G.; Adojaan, K.; Vizzini, A.; Suija, A.; Pfister, D.; Baral, H.-O.; Järv, H.; Madrid, H.; Nordén, J.; Liu,

J.-K.; Pawlowska, J.; Põldmaa, K.; Pärtel, K.; Runnel, K.; Hansen, K.; Larsson, K.-H.; Hyde, K. D.; Sandoval-Denis, M.; Smith, M. E.; Toome-Heller, M.; Wijayawardene, N. N.; Menolli, N.; Reynolds, N. K.; Drenkhan, R.; Maharachchikumbura, S. S. N.; Gibertoni, T. B.; Læssøe, T.; Davis, W.; Tokarev, Y.; Corrales, A.; Soares, A. M.; Agan, A.; Machado, A. R.; Argüelles-Moyao, A.; Detheridge, A.; de Meiras-Ottoni, A.; Verbeken, A.; Dutta, A. K.; Cui, B.-K.; Pradeep, C. K.; Marín, C.; Stanton, D.; Gohar, D.; Wanasinghe, D. N.; Otsing, E.; Aslani, F.; Griffith, G. W.; Lumbsch, T. H.; Grossart, H.-P.; Masigol, H.; Timling, I.; Hiiesalu, I.; Oja, J.; Kupagme, J. Y.; Geml, J.; Alvarez-Manjarrez, J.; Ilves, K.; Loit, K.; Adamson, K.; Nara, K.; Küngas, K.; Rojas-Jimenez, K.; Bitenieks, K.; Irinyi, L.; Nagy, L. G.; Soonvald, L.; Zhou, L.-W.; Wagner, L.; Aime, M. C.; Öpik, M.; Mujica, M. I.; Metsoja, M.; Ryberg, M.; Vasar, M.; Murata, M.; Nelsen, M. P.; Cleary, M.; Samarakoon, M. C.; Doilom, M.; Bahram, M.; Hagh-Doust, N.; Dulya, O.; Johnston, P.; Kohout, P.; Chen, Q.; Tian, Q.; Nandi, R.; Amiri, R.; Perera, R. H.; dos Santos Chikowski, R.; Mendes-Alvarenga, R. L.; Garibay-Orijel, R.; Gielen, R.; Phookamsak, R.; Jayawardena, R. S.; Rahimlou, S.; Karunarathna, S. C.; Tibpromma, S.; Brown, S. P.; Sepp, S.-K.; Mundra, S.; Luo, Z.-H.; Bose, T.; Vahter, T.; Netherway, T.; Yang, T.; May, T.; Varga, T.; Li, W.; Coimbra, V. R. M.; de Oliveira, V. R. T.; de Lima, V. X.; Mikryukov, V. S.; Lu, Y.; Matsuda, Y.; Miyamoto, Y.; Kõljalg, U.; Tedersoo, L. FungalTraits: A User-Friendly Traits Database of Fungi and Fungus-like Stramenopiles. *Fungal Divers* **2020**, *105* (1), 1–16. https://doi.org/10.1007/s13225-020-00466-2.

(10) Davis, N. M.; Proctor, Di. M.; Holmes, S. P.; Relman, D. A.; Callahan, B. J. Simple Statistical Identification and Removal of Contaminant Sequences in Marker-Gene and Metagenomics Data. *Microbiome* **2018**, *6* (1), 1–14. https://doi.org/10.1186/S40168-018-0605-2/FIGURES/6.

(11) Li, J.; Xu, W.; You, B.; Sun, Y. Dynamic Variations of Ammonia in Various Life Spaces and Seasons and the Influences of Human Activities. *Build Environ* **2022**, *212*, 108820. https://doi.org/10.1016/j.buildenv.2022.108820.

(12) Yang, S.; Bekö, G.; Wargocki, P.; Zhang, M.; Merizak, M.; Nenes, A.; Williams, J.; Licina, D. Physiology or Psychology: What Drives Human Emissions of Carbon Dioxide and Ammonia? *Environ Sci Technol* **2024**, *58* (4), 1986–1997. https://doi.org/10.1021/acs.est.3c07659.

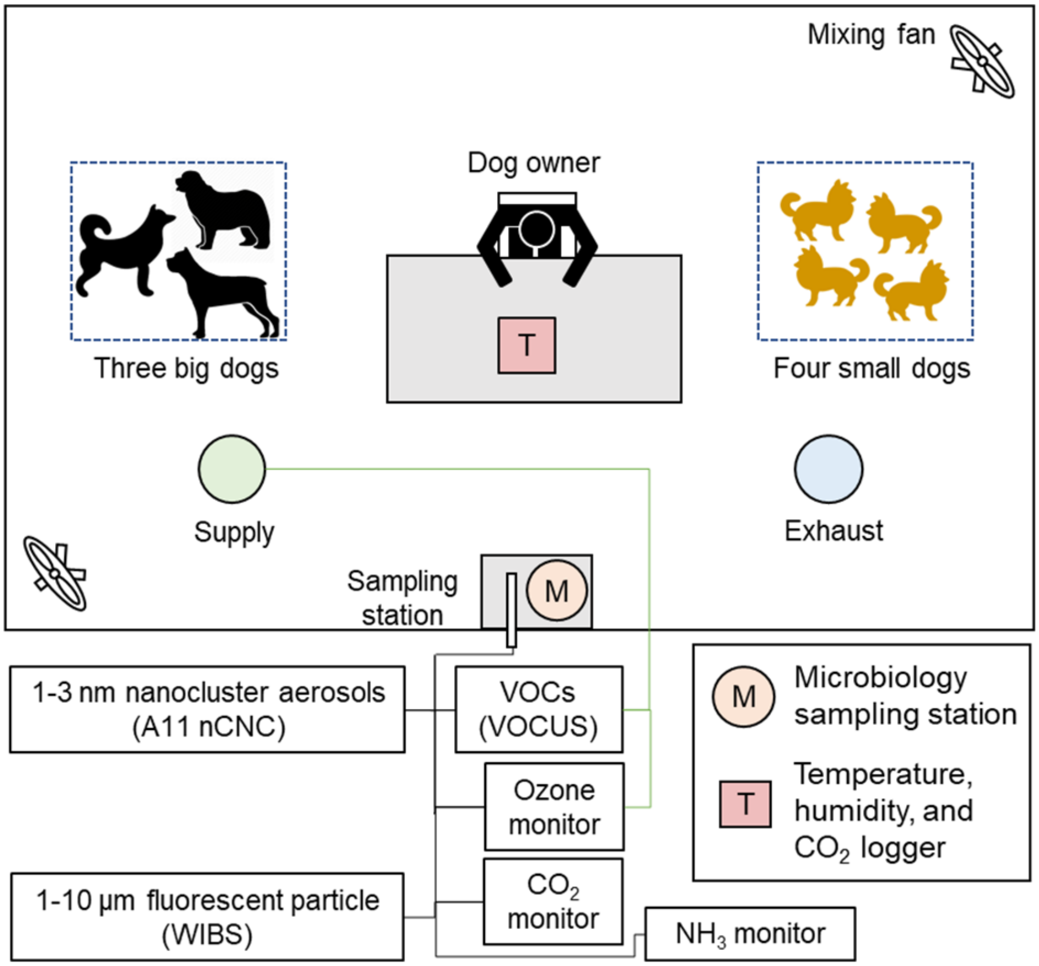

Fig. S1.

Schematic layout of the chamber setup. The chamber had a volume of 62 m^3 (Length 5.3 m × Width 4.3 m × Height 2.5 m). The chamber wall was made of stainless steel, and the ceiling was covered by aluminum foil, whereas the floor was covered by vinyl plates. We performed three sets of experiments in the chamber: (1) One dog owner only; (2) Four small dogs and their owner; and (3) Three big dogs and their owner.

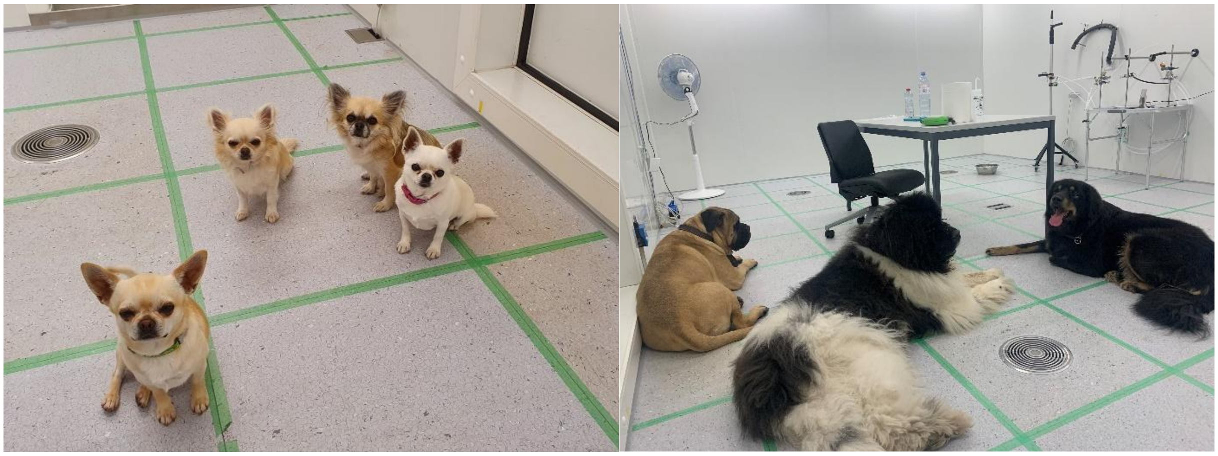

Fig. S2.

Four small dogs (left) and three big dogs (right) inside the chamber during experiments.

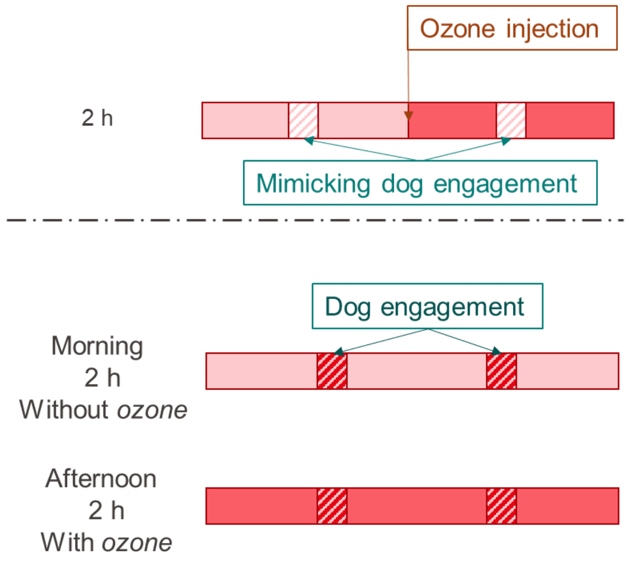

Fig. S3.

Experiment procedure for owner only (upper, D0) and dogs and owner together (lower, D1-D4)

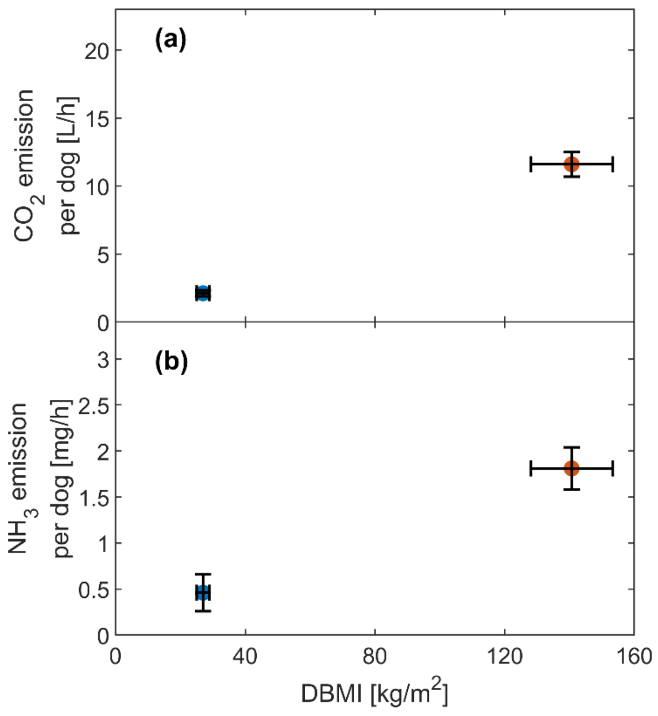

Fig. S4.

Relationship between the DBMI and dogs' (a) CO_2 and (b) NH_3 emission rates. Vertical and horizontal bars represent standard deviations of emission rates and dog BMI respectively. DBMI (dog body mass index) was calculated as dog's weight divided by the square of dog's withers height (Table S2).

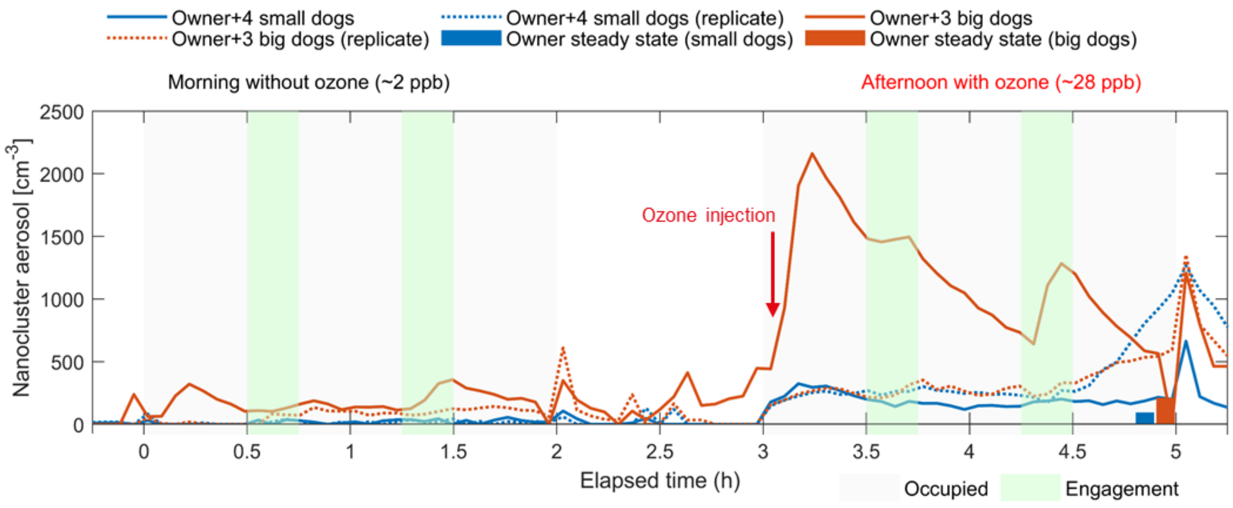

Fig. S5.

1-3 nm nanocluster aerosol emissions from dogs and their owners. Gray shaded area represents occupied periods, whereas green shaded area corresponds to dog engagement events. The bars represent nanocluster aerosol concentrations at the end of the owner-only experiments with ozone present (quasi-steady state).

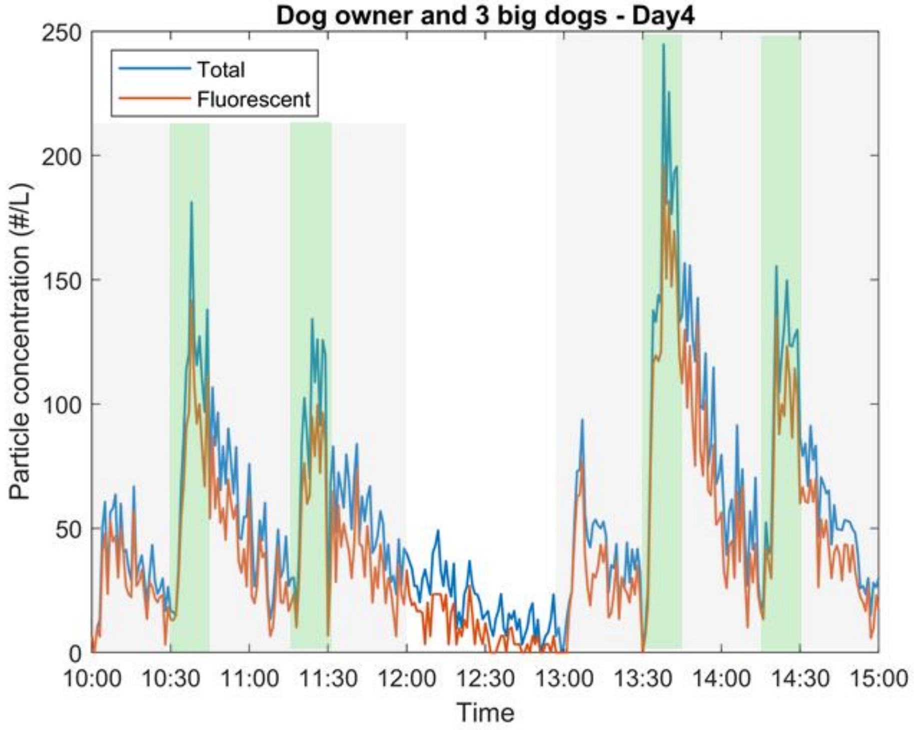

Fig. S6.

An example of 1-10 μm total and fluorescent particle concentrations during the morning and afternoon session. Data are from D4 with dog owner and three big dogs. Gray shaded area represents occupied periods, whereas green shaded area corresponds to dog engagement events.

Table S1. Detailed information about the two dog groups

Group	Breed	Height (cm)*	Weight (kg)	"DBMI" (kg/m^2)
Small dogs	Chihuahua	32	2.5	24.4
		35	3.5	28.6
		35	3.5	28.6
		32	2.7	26.4
Big dogs	Tibetan Mastiff	57	45	138.5
	Newfoundland	64	53	129.4
	Mastiff	72	80	154.3

* Refers to withers height: the vertical distance from the ground to the highest point of the shoulder blades at a standing position.

Table S2. Experimental schedule

Exp. No.	Condition
D0	1 Dog owner<br>(Morning for the owner of big dogs and afternoon for the owner of small dogs)
D1	4 Small dogs & 1 Dog owner (Morning without ozone and afternoon with ozone)
D2	4 Small dogs & 1 Dog owner (Replicate of D1)
D3	3 Big dogs & 1 Dog owner (Morning without ozone and afternoon with ozone)
D4	3 Big dogs & 1 Dog owner (Replicate of D3)

Table S3. Emission rates of main VOCs emitted from dogs and comparison with their owners.

Without ozone injection (~2 ppb ozone)				
		Signal	Emission rate (μg/h/dog)	Dog-to-human ratio
Small dog	Quantified with calibration	MVK *	5.3	0.7
		Pentanal *	45.7	2.8
	Tentatively identified without calibration	$C_4H_{11}O_2^+$	32.8	0.6
		$C_8H_{11}O_2^+$	3.4	2.8
Big dog	Quantified with calibration	Acetone *	349.6	1.1
		6MHO *	7.8	1.4
		Nonanal *	43.9	1.4
	Tentatively identified without calibration	$C_5H_{11}O_2^+$	59.4	15.4
		$C_6H_5O_2^+$	9.3	5.8
		$C_8H_9O^+$	1.2	0.6
		$C_8H_{17}O_2^+$	1.5	0.4
		$C_9H_{15}^+$	1.6	0.4
		Decanal	5.3	0.7
		$C_{12}H_{25}^+$	1.2	0.1
		$C_{13}H_{21}^+$	1.1	0.6
		$C_{13}H_{23}O^+$	1.9	0.7
With ozone injection (~28 ppb ozone)				
		Signal	Emission rate (μg/h/dog)	Dog-to-human ratio
Small dog	Quantified with calibration	MVK *	2.9	0.3
		4OPA *	35.8	0.5
		Pentanal *	21.1	0.2
		Heptanal *	35.4	0.2
		6MHO *	15.3	0.1
		Octanal *	35.8	0.2
	Tentatively identified without calibration	$C_4H_7O_2^+$	6.4	2.9
		$C_5H_7O^+$	34.2	0.5
		$C_5H_9O_3^+$	2.9	0.5
		$C_6H_{15}O_2^+$	2.3	0.6
		$C_7H_{13}O^+$	1.8	0.2
		$C_7H_{15}O_2^+$	2.3	0.2
		$C_8H_{11}O_2^+$	1.4	1.7
		$C_8H_{13}O^+$	1.3	0.3
		$C_8H_{17}^+$	1.3	0.1
		$C_8H_{17}O_2^+$	3.9	0.3
		$C_9H_{15}O_2^+$	2.1	0.3
		$C_{10}H_{15}O^+$	1.1	0.3
		$C_{10}H_{17}O_2^+$	0.7	0.2
		Decanal	15.7	0.3
		Undecanal	2.6	0.2

		$C_{15}H_{23}^+$	1.9	0.6
Big dog	Quantified with calibration	Acetone *	273.8	0.6
		MVK *	1.5	0.1
		4OPA *	27.1	0.4
		Heptanal *	224.5	0.6
		6MHO *	22.7	0.3
		Octanal *	17.9	0.3
		Nonanal *	131.5	0.4
	Tentatively identified without calibration	$C_5H_7O^+$	26.0	0.4
		$C_5H_9^+$	45.6	0.2
		$C_5H_{11}O_2^+$	30.4	3.5
		$C_6H_9^+$	4.0	0.4
		$C_7H_{11}^+$	1.7	0.3
		$C_7H_{15}O_2^+$	2.3	0.5
		$C_8H_9O^+$	3.4	0.7
		$C_8H_{13}O^+$	1.8	0.4
		$C_8H_{15}O_2^+$	3.1	0.5
		$C_8H_{17}^+$	2.5	0.7
		$C_8H_{17}O_2^+$	3.7	0.8
		$C_9H_{15}O_2^+$	2.2	0.5
		$C_9H_{19}O_2^+$	4.5	0.7
		$C_{10}H_{13}^+$	0.7	0.7
		$C_{10}H_{15}O^+$	1.8	1.4
		$C_{10}H_{15}O_2^+$	0.7	0.6
		$C_{10}H_{17}O_2^+$	1.2	0.5
		$C_{10}H_{19}O^+$	1.6	2.8
		$C_{10}H_{19}O_3^+$	1.0	0.4
		Decanal	17.5	0.4
		Undecanal	2.4	0.4
		Dodecanal	1.8	0.4
		$C_{13}H_{21}^+$	1.5	1.0
		$C_{13}H_{23}O^+$	2.2	0.7
		$C_{13}H_{27}O^+$	0.9	0.4

* These compounds have specific calibration factors and thus accurate values of emission rate. The reported emission rates of other compounds should be applied with caution, as they do not have individual calibrations. Nevertheless, the dog-to-human ratios are reliable. Because all species were measured with the same instrument settings, any calibration biases or fragmentation uncertainties are expected to affect the dog and human signals proportionally. Thus, while the absolute magnitudes carry uncertainty, the relative differences between dogs and humans are reliable.
